# High-Grade Hepatic Spindle Cell Carcinoma as a Cause of Diffuse Right Hepatic Lobe Hypermetabolism Identified With 18F-Fluorodeoxyglucose Positron Emission Tomography/Computed Tomography (18F-FDG PET/CT) Imaging

**DOI:** 10.7759/cureus.68793

**Published:** 2024-09-06

**Authors:** Dhuha Al-Adhami, Marwa Al-shatti, Kamal Alrabi, Mohammad Haidar, Akram Al-Ibraheem

**Affiliations:** 1 Nuclear Medicine and Positron Emission Tomography/Computed Tomography (PET/CT), King Hussein Cancer Center (KHCC), Amman, JOR; 2 Pathology, King Hussein Cancer Center (KHCC), Amman, JOR; 3 Medical Oncology, King Hussein Cancer Center (KHCC), Amman, JOR; 4 Nuclear Medicine, American University of Beirut Medical Center, Beirut, LBN

**Keywords:** 18f-fdg, hepatic superscan, hepatocellular sarcoma, pet/ct, spindle cell carcinoma

## Abstract

We present a rare case of high-grade spindle cell liver carcinoma investigated using ^18^F-fluorodeoxyglucose positron emission tomography/computed tomography (^18^F-FDG PET/CT) in a 70-year-old man with worsened clinical symptoms for more than three months. These include hiccups, belching, loss of appetite, and significant weight loss of 30 kg over the last three months. ^18^F-FDG PET/CT was employed as part of the initial diagnostic assessment, revealing intense diffuse right hepatic lobe hypermetabolism. Fused PET/CT images revealed a locally confined right hepatic lobe tumor exhibiting heterogeneous metabolic activity with predominant hypermetabolism surrounding two internally contained hypometabolic islands. These findings suggested an aggressive and unusual neoplastic pattern. Ultrasound-guided biopsy confirmed high-grade spindle cell sarcoma of the liver. Unfortunately, the patient passed away shortly thereafter, precluding therapeutic decisions.

## Introduction

Spindle cell hepatocellular carcinoma (SpHCC) is a rare and aggressive neoplasm, accounting for less than 1% of HCC cases and predominantly affecting elderly males [1]. These tumors typically present as biphasic, consisting of both epithelial and sarcomatous cells, and most commonly affect the right lobe of the liver [2]. SpHCC is believed to develop sporadically or frequently as a secondary condition following therapy for pre-existing HCC [3].

Advancements in imaging techniques have significantly improved the detection of various neoplasms [4]. One notable example is ^18^F-Fluorodeoxyglucose positron emission tomography/computed tomography (^18^F-FDG PET/CT), which serves as a comprehensive tumor imaging agent, facilitating the evaluation of various neoplasms [5]. Despite its utility, not all tumors express ^18^F-FDG; for instance, HCC typically exhibits low ^18^F-FDG uptake, especially in well-differentiated tumors, due to high glucose-6-phosphatase activity and low expression of glucose transporters [6]. However, certain rare HCC subtypes, such as those with sarcomatous elements, behave differently. Sarcomatous HCC, a rare and aggressive subtype, is characterized by higher ^18^F-FDG uptake, reflecting its aggressive nature, higher mutation rates, and poorer prognosis compared to non-sarcomatous types [7,8].

To our knowledge, this case presents the first instance of ^18^F-FDG PET/CT imaging in a patient diagnosed with SpHCC. The scan revealed an intense diffuse hypermetabolism in the right hepatic lobe, thereby underscoring the aggressive nature of the disease.

## Case presentation

**Figure 1:**
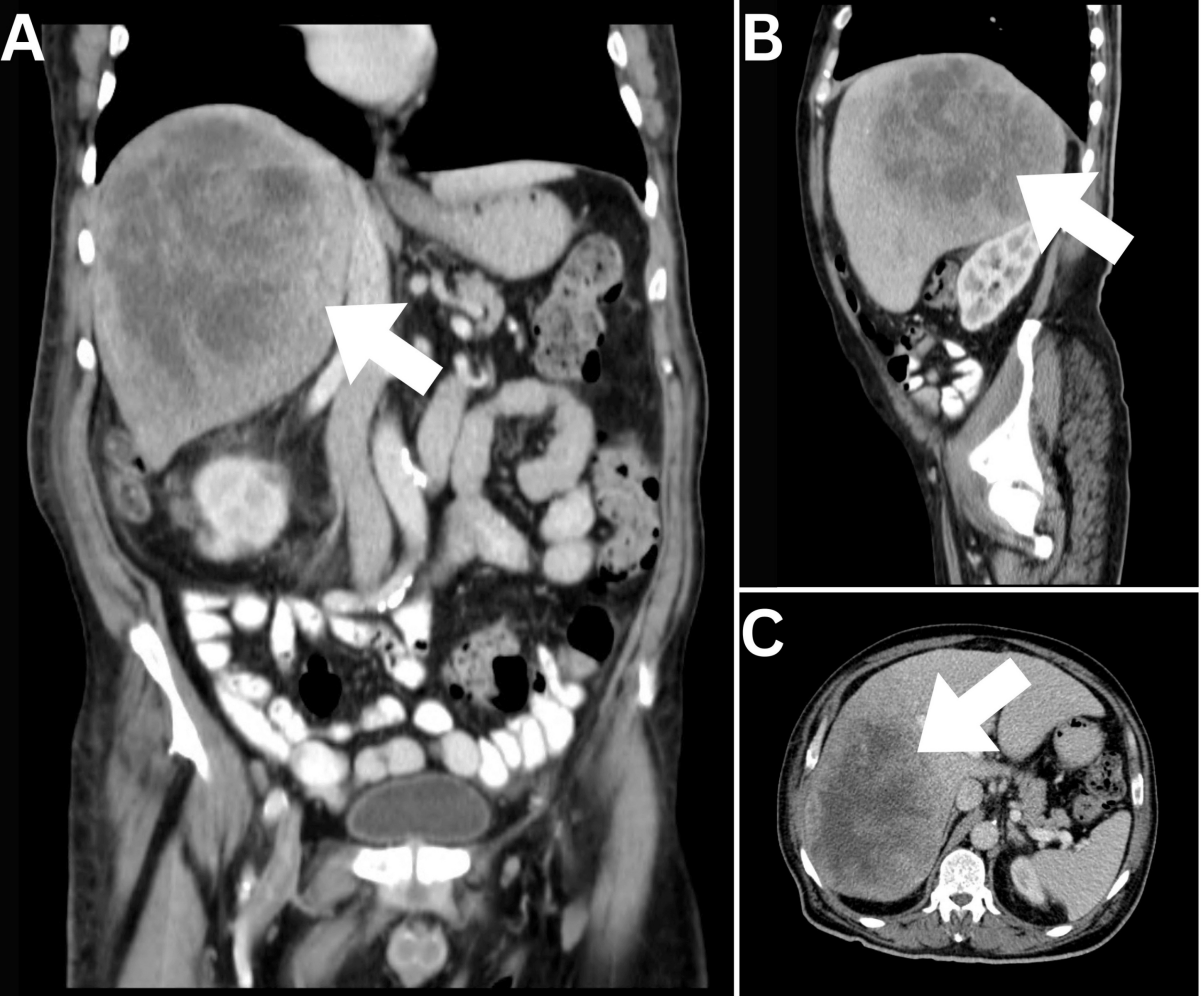
Abdominopelvic CT (A) Coronal, (B) Sagittal, and (C) Axial views of abdominopelvic CT demonstrated a large complex density mass lesion occupying a major part of the right hepatic lobe (arrows).

A 70-year-old man presented with a complicated medical history consisting of diabetes mellitus, hypertension, ischemic heart disease, and dyslipidemia. He had a negative past surgical history. The patient's presenting complaints began three months prior to the consultation, with symptoms including hiccups, belching, loss of appetite, significant weight loss of 30 kg over the last three months, fatigue, vomiting twice daily, and postural dizziness. Laboratory investigations revealed severe anemia with a hemoglobin level of 6.6 g/dl, which had been treated with a blood transfusion two weeks prior to the visit. Initial diagnostic imaging was performed and included an abdominopelvic computed tomography (CT) scan, which revealed a large, rounded soft tissue mass occupying major parts of the right hepatic lobe (Figure 1).

**Figure 2:**
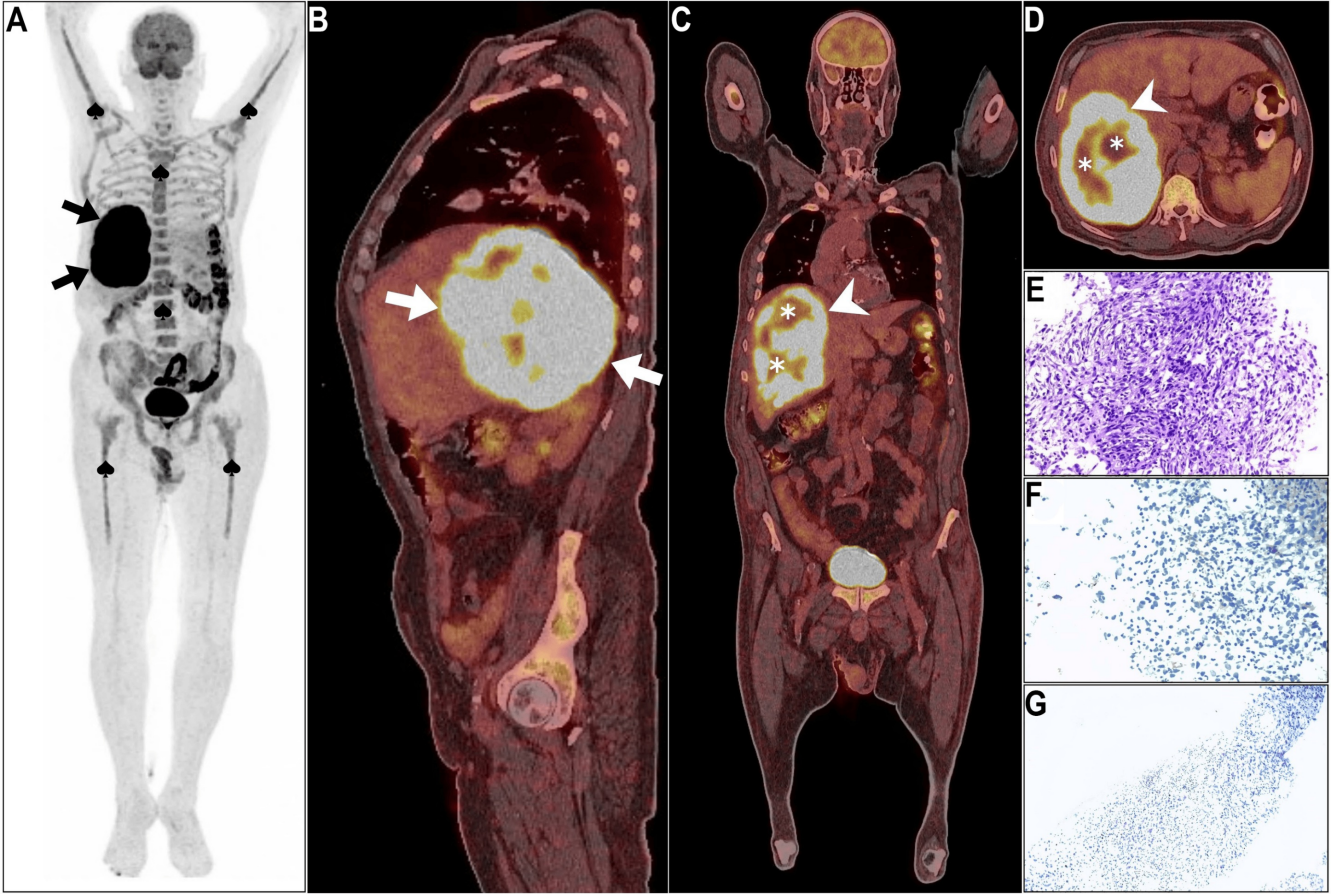
18F-FDG PET/CT study (A) Maximum intensity projection image showed intense right hepatic lobe hypermetabolism (arrows), with suppressed brain activity, and diffuse bone marrow ^18^F-FDG activity linked to anemia (spades). (B-D) sagittal, coronal, and axial PET/CT images revealed the tumor's extent and heterogeneity, with intense overall ^18^F-FDG uptake and two photopenic islands (asterisks). (E) Histopathology identified anaplastic spindle-shaped cells with pleomorphic nuclei, while (F, G) epithelial membrane antigen, and pancytokeratin immunostains displayed negative expression and excluded epithelial origin.

This mass exhibited areas of necrosis and was not highly vascular, suggesting a malignant etiology. The patient was transferred to our cancer center. Comprehensive laboratory tests, including complete blood count, liver function test, and renal function test, were performed. These findings were unremarkable apart from markedly elevated aspartate aminotransferase (509 U/L), alanine aminotransferase (403 U/L), and alkaline phosphatase (514 U/L), in addition to moderate anemia (hemoglobin of 8.2 g/dL). Concurrently, an ultrasound-guided biopsy was conducted for histopathological analysis. Pending histopathological results, ^18^F-FDG PET/CT was performed (Figure 2).

Maximum intensity projection (MIP) image revealed intense right hepatic lobe hypermetabolism with physiologic ^18^F-FDG uptake suppression in the brain (Figure 2A, arrows). In addition, reactive diffuse homogeneous bone marrow activity was noted involving the whole axial and proximal peripheral skeleton to correlate with ongoing anemia in the patient (Figure 2A, spades). The sagittal PET/CT image depicted the extent of the tumor within the right hepatic lobe (Figure 2B, arrows). Coronal and axial PET/CT images highlighted the heterogeneous nature of the tumor, with marked overall ^18^F-FDG expression (Figure 2C, 2D; arrows), containing two islands of hypometabolism indicative of necrosis (Figure 2C, 2D; asterisks). The maximum standardized uptake value (SUVmax) for the tumor of interest was 34.2 (Figure 2C, 2D; arrows). Overall, the results indicated the complex, aggressive, and atypical nature of the primary tumor, which was confined to the right hepatic lobe. Histopathologic examination revealed that the tumor consisted of anaplastic spindle-shaped cells with arid interlacing bundles showing a partial storiform pattern and hosting pleomorphic nuclei (Figure 2E). Both epithelial membrane antigen and pancytokeratin immunostains displayed negative expression. Therefore, immunohistochemistry analysis excluded the epithelial nature of the tumor (Figure 2F, 2G). Despite establishing a diagnosis of stage II high-grade spindle cell carcinoma of the liver, subsequent therapy decisions were postponed due to worsening clinical conditions, unconsciousness, and transfer to the intensive care unit, leading to death from hepatic failure two weeks after admission.

## Discussion

Upon reviewing the MIP image, diffuse and intense right hepatic lobe ^18^F-FDG localization is evident. This pattern has been previously documented using ^18^F-FDG PET/CT imaging. Typically, this phenomenon affects both liver lobes, leading to the suppression of ^18^F-FDG physiological uptake in active sites such as the brain, urinary bladder, and kidneys.

Basu and Nair were among the first to describe this imaging pattern in hematologic malignancies, coining the term "hepatic superscan" in this context [9]. This imaging pattern differed significantly from the typical focal concentration of ^18^F-FDG seen in metastatic liver involvement. The diffuse hepatic uptake served as the earliest indicator of extensive hepatic involvement by Hodgkin's disease, crucial for diagnosing hepatic involvement that might otherwise have been missed using conventional imaging techniques [9]. Hepatic superscan was inspired by a similar appearance to a "superscan" seen in conventional skeletal scintigraphy, where there is intense, diffuse uptake in the bones with relatively low soft tissue uptake [10]. Since this initial observation, numerous cases involving various neoplasms have been reported [11-16]. The etiologies of hepatic superscan are diverse, encompassing neoplastic (malignant or metastatic) and inflammatory (primarily granulomatous disease) causes [11-16]. Our case closely mirrors the general appearance of a hepatic superscan, with the notable exception that hepatic hypermetabolism was confined to the right hepatic lobe, where the primary SpHCC is located. Furthermore, axial fused images in our case revealed a complex heterogeneous ^18^F-FDG pattern characterized by the presence of two photopenic islands. These intra-tumoral photopenic areas may indicate tumor necrosis, histopathologic heterogeneity, or a hypoxic state [17].

It is crucial to recognize that this molecular imaging pattern has several implications beyond its distinctive characteristics. Previous reports have associated it with poor prognostic outcomes [12,16]. Additionally, such intense ^18^F-FDG uptake may signify an aggressive underlying disease. In some instances, observing such an imaging finding can be followed by rapid and sudden demise even before initiating any therapeutic intervention. For example, Abdlkadir et al. reported a case of hepatic superscan caused by fulminant hepatic metastasis from newly diagnosed medullary thyroid carcinoma [16]. After two months of right upper quadrant pain and jaundice, initially attributed to benign pathologies, the patient was referred to a tertiary cancer institute where ^18^F-FDG PET/CT highlighted the hypermetabolic primary tumor alongside hepatic superscan. An imaging-guided biopsy was performed for histopathologic analysis. Unfortunately, the patient experienced a rapid demise even before the biopsy results were available [16]. Similarly, in our case, the patient also had a quick demise, underscoring the aggressive and poor prognostic nature of diffuse hepatic hypermetabolism.

## Conclusions

This case represents the first instance of imaging for SpHCC exhibiting diffuse intense hypermetabolism in the right hepatic lobe, underscoring the aggressive nature of this sarcomatous HCC subtype. It also highlights the significance of ^18^F-FDG PET/CT in staging, disease prognostication, and identifying atypical complex patterns. Furthermore, this case supports the applicability of ^18^F-FDG PET/CT in sarcomatous subtypes of HCC. Diffuse hepatic hypermetabolism observed on ^18^F-FDG imaging could be indicative of severe hepatic involvement due to an aggressive pathological condition and may portend a poor prognosis. This phenomenon may result from either an inflammatory primary malignancy or a metastatic process. As a consequence, such molecular imaging findings should invariably prompt histopathological examination to rule out significant pathology. Furthermore, the systematic documentation and investigation of similar cases could contribute to the validation of these observations and facilitate the establishment of a definitive conclusion in the future.
